# A urinary proteomic study in hypercalciuric dogs with and without calcium oxalate urolithiasis

**DOI:** 10.14202/vetworld.2022.2937-2944

**Published:** 2022-12-27

**Authors:** Sumonwan Chamsuwan, Chollada Buranakarl, Kris Angkanaporn, Thasinas Dissayabutra, Natthaya Chuaypen, Trairak Pisitkun, Nuttiya Kalpongnukul

**Affiliations:** 1 Department of Physiology, Faculty of Veterinary Science, Chulalongkorn University, Bangkok, Thailand; 2 Metabolic Disease in Gut and Urinary System Research Unit, Department of Biochemistry, Faculty of Medicine, Chulalongkorn University, Bangkok, Thailand; 3 Center of Excellence in Systems Biology, Research Affairs, Faculty of Medicine, Chulalongkorn University, Bangkok, Thailand

**Keywords:** calcium oxalate stone, dog, hypercalciuria, urinary biomarker

## Abstract

**Background and Aims:**

Hypercalciuria is an important predisposing factor commonly found in humans and dogs with calcium oxalate (CaOx) urolithiasis. Calcium oxalate crystals can induce an inflammatory reaction that subsequently produces several proteins that have an inhibitory or stimulatory effect on stone formation. This study aimed to evaluate the differences in urinary proteomic profiles between hypercalciuric CaOx stone dogs and hypercalciuric stone-free dogs (CaOx stone and control groups, respectively).

**Materials and Methods:**

Seven dogs with hypercalciuric CaOx urolithiasis and breed-, sex-, and aged-matched controls with hypercalciuria were included in the study. Serum and urine samples were obtained from all dogs to analyze electrolytes. Urinary proteomic profiles were analyzed using liquid chromatography-mass spectrometry. Student’s t-test was used to compare the differences between groups.

**Results:**

Forty-nine urinary proteins were identified in the stone-free and CaOx stone groups, whereas 19 and 6 proteins were unique in the CaOx stone and stone-free groups, respectively. The urinary thrombomodulin level was significantly higher in the CaOx stone group (relative ratio = 1.8, p < 0.01) than in the stone-free group.

**Conclusion:**

This study demonstrated that urinary proteomic profiles may be used as a candidate biomarker for urinary tract injury in CaOx urolithiasis in dogs.

## Introduction

Urolithiasis is a common urologic disease with increased incidence over time in both humans and dogs [1]. Calcium oxalate (CaOx) stone is the most common type of stone in humans and among the top in dogs [1, 2]. A common treatment of stone is surgical removal with dietary modification. However, the recurrent rate is still high, approximately 48%–57% within 3 years in dogs [3]. The high recurrence is believed to be due to uncorrected and unidentified underlying diseases. In dogs, hypercalciuria is the most common metabolic abnormality found in urolithiasis [4, 5]. In addition, elevated urinary calcium excretion increases urine supersaturation, facilitating CaOx crystallization, and stone formation [6]. Nevertheless, excessive urinary calcium excretion could be found in normal and CaOx stone-forming dogs and cannot be used to identify the risk of urolithiasis.

Cellular injury and inflammation are associated with CaOx stone pathogenesis in humans [7]. These processes involve the production of several macromolecules, especially proteins that are responsible for matrix formation, inflammatory response, oxidative stress, fibrogenesis, and regeneration [8, 9]. In addition, intermediate proteins in cellular injury and apoptotic or healing pathways should be expressed [10, 11]. Specific proteins found in these processes might be associated with the prognosis of the disease or can be used as a biomarker for CaOx stone formation.

In humans, proteomic studies are frequently used to identify the culprit proteins of a disease. In urolithiasis, certain studies have reported several proteins, such as the Tamm–Horsfall protein, transferrin, albumin, and some inflammatory cytokines that were related to the risk of CaOx stone formation [11–13]. However, data on urine proteome in dogs with urolithiasis are very limited [14, 15].

This study aimed to conduct proteomic analyses to identify the urine proteins associated with urolithiasis in dogs with hypercalciuria to evaluate the risk of lithogenesis and recurrence. We believe that our finding could demonstrate the pathogenesis of stone formation and might be used as a targeted treatment for urolithiasis.

## Materials and Methods

### Ethical approval and Informed consent

Animal ethics approval was obtained from the Chulalongkorn University Animal Care and Use Committee, Faculty of Veterinary Science, Chulalongkorn University (Protocol number 1831101), Bangkok, Thailand. Informed consent was obtained from the owners.

### Study period and location

The study was conducted from February 2019 to January 2020 at the Small Animal Hospital, Chulalongkorn University, Bangkok, Thailand.

### Sample population

The study included 14 dogs with a hypercalciuric condition, that is, defined as urine calcium-to-creatinine ratio (UCa/Cr) of ≥ 0.05 as described by Groth *et al*. [16]. The stone-free groups consisted of breed-sex-, and age (within 2 years)-matched dogs (n = 7) and had no history of uroliths confirmed by radiography or ultrasonography. The CaOx stone group (n = 7; stone-former) was diagnosed with CaOx stone disease at any episode, and a stone was removed in the recent 12 weeks. The stone composition was analyzed by polarizing light microscopy and infrared spectroscopy at the Minnesota Urolith Center, University of Minnesota, and the major component of uroliths was CaOx (≥70%). The stone-free group (stone-free) consumed a regular commercially available adult diet. In contrast, the CaOx stone group consumed a prescription diet for controlling urolithiasis (Canine urinary SO, Royal Canin Veterinary Diet, Waltham Centre for Pet Nutrition, USA or c/d Canine Prescription Diet, Hill’s Pet Nutrition Inc., USA). Dogs with remnant stones detected by either abdominal radiography or ultrasonography at the time of study recruitment were excluded. No dogs with active urinary tract infection, as determined by urinalysis were recruited.

### Sample collection

Before the experimental study, feed was withheld for at least 8 h from all dogs, but they had free access to water. Approximately 3 mL of blood was collected into a heparinized tube. The plasma was separated immediately, and calcium and creatinine concentrations were measured. Some plasma samples were stored at −20°C for later analysis of magnesium (Mg) concentration.

Approximately 10 mL of urine was obtained by either voiding or cystocentesis for the determination of calcium and creatinine concentration. Some urine samples were kept at −20°C for analysis of Mg concentration. Some aliquots were stored at −80°C for proteomic analysis.

### Analysis of plasma and urine electrolytes

Plasma and urinary concentrations of Ca were measured using an automated analyzer (The IL ILab 650 Chemistry Analyzer, Diamond Diagnostic, MA, USA). In contrast, Mg concentration was measured by inductively coupled plasma optical emission spectrometry (Optima 5400, Perkin Elmer Optima, Waltham, MA, USA). Plasma and urinary creatinine levels were determined using an enzymatic method by an automated analyzer (The IL ILab 650 Chemistry Analyzer, Diamond Diagnostic). The urinary excretion rate is presented as per creatinine ratio. In each sample, protein concentration was measured by the Bradford assay [17]. The urine protein-to-creatinine (UPC) ratio was calculated in each dog by dividing the urine concentrations of protein and creatinine.

### Sample preparation for the proteomic analysis

The volume of urine sample loading from each dog was calculated based on the UPC ratio. The amount of individual protein was expressed in relation to the total protein in each sample. Urine proteins were extracted by methanol precipitation and separated by one-dimensional sodium dodecyl sulfate-polyacrylamide gel electrophoresis (SDS-PAGE).

For in-gel digestion, the gel was cut into small pieces. Then, 200 μL of 25 mM ammonium bicarbonate and 100% acetonitrile (ACN) were added for the SDS removal and Coomassie staining. Proteins were reduced by 10 mM dithiothreitol in 25 mM ammonium bicarbonate at 56°C for 45 min, followed by alkylation with 10 mM iodoacetamide for 30 min at room temperature in the dark room. The supernatant was discarded. Then, 100 μL of 25 mM ammonium bicarbonate in 50% ACN was added to wash the gels for 10 min. The solvent was removed, and 200 μL of 100% ACN was added to shrink the gel pieces and dried in a speed vacuum for 10 min. Fresh trypsin (10 ng/μL) was added to cover the gel pieces and incubated on ice at 4°C for 60 min. Ammonium bicarbonate was added to the sample and incubated at 37°C overnight. Then, 0.1% formic acid (FA) was added for peptide extraction; this procedure was performed twice. The collected supernatant was dried using a speed vacuum and kept at −80°C. On the day of analysis, the peptide extract was resuspended in 0.1% FA and loaded onto Jupiter C18 filter column.

### Liquid chromatography-mass spectrometry (LC-MS/MS) and data analysis

The peptides were separated and analyzed by a nanoflow liquid chromatograph (EASY-nLC 1000 Liquid Chromatograph, Thermo Fisher Scientific, MA, USA) coupled to a mass spectrometer (Q Exactive™ Plus Hybrid Quadrupole-Orbitrap™ Mass Spectrometer, Thermo Fisher Scientific, MA, USA) through EASY-Spray™ Sources (Thermo Fisher Scientific, MA, USA). The peptides were eluted with the following ACN gradients in 0.1% FA: 5%–20% for 60 min, 20%–40% for 20 min, 40%–98% for 2 min, and 98%–100% for 8 min, at a flow rate of 300 nL/min. Orbitrap precursor spectra were collected from 350 to 1400 m/z for 90 min at a resolution of 70,000 with AGC target at 3 × 106 ions. The top 10 most abundant precursors with 2+ to 4+ charge states were selected and fragmented by higher-energy C-trap dissociation to generate MS/MS data. A dynamic exclusion of 30 s was used.

Raw data files from the mass spectrometer were used to search against a canine protein database using SEQUEST HT™ database-searching algorithms, and mass tolerances for precursor and fragment ions were set to 10 ppm and 0.02 Da. The Percolator algorithm was used to compute the false-positive discovery rate of the identified peptides based on Q-values 1% using Proteome Discoverer software (Thermo Scientific™ Proteome Discoverer™ software v.2.1.1.21, Thermo Fisher Scientific, MA, USA). The protein sequences and functional information of specific proteins were identified as annotated in the UniProtKB/Swiss-Prot database (https://www.uniprot.org/).

### Statistical analysis

Data are presented as mean ± standard error. For proteomic analysis, data of unique proteins were transformed in logarithms. The relative ratio or fold-change of each protein was compared between the stone-free group and the CaOx stone group. The unpaired t-test was used to compare the mean of variables, including serum and urine electrolytes, UPC ratio, urine pH, urine-specific gravity, and differences in protein abundances between groups. All analyses were performed using the IBM SPSS Statistics for Windows version 22 (IBM Corp., Armonk, NY, USA), and p < 0.05 was set as statistically significant.

## Results

### Patient’s characteristics

Seven dogs in both groups were recruited, including five Pomeranians, one Chihuahua, and one Miniature Schnauzer. Both the stone-free and CaOx stone groups were composed of six males and one female dog. The number of intact male, castrated male, and spayed female dogs in the stone-free and CaOx stone groups were 3, 3, and 1 vs. 4, 2, and 1, respectively. The average age of the dogs in the stone-free group was not different from that of the CaOx stone group (7.3 ± 0.8 vs. 6.4 ± 0.9, p = 0.48). Urine samples from 1 and 6 dogs in the CaOx stone group and 5 and 2 dogs in the stone-free group were collected by voiding and cystocentesis, respectively. In the CaOx stone group, the average time of urine sample collection was 14.7 ± 1.6 weeks after cystotomy.

### Serum and urine electrolytes

All dogs had serum calcium, Mg, and creatinine levels within the reference range. All serum parameters, including calcium, Mg, and creatinine were not different between the CaOx stone group and stone-free group (Table-1). Regarding urinary excretion rate, no differences in UCa/Cr, UMg/Cr, UPC ratio, and urine-specific gravity were found between the CaOx stone and stone-free groups. However, the CaOx stone group had lower urine pH than the stone-free group (p < 0.05). The microscopic examination of urine sediment showed no evidence of CaOx crystals in the stone-free group. In contrast, some CaOx crystals were found in all dogs in the CaOx stone group. Moreover, no evidence of hematuria was found in all urine samples of both groups as determined by both the urine strip test and microscopic examination of urine sediment.

**Table-1:** Serum and urine electrolytes in study population.

Variables	Control (n = 7)	Case (n = 7)	p-value
Serum electrolytes (mg/dl)			
Calcium	8.60 ± 0.46	7.86 ± 0.66	0.37
Magnesium	2.32 ± 0.18	1.99 ± 0.17	0.21
Creatinine	0.80 ± 0.08	0.74 ± 0.11	0.68
Urinary excretion of electrolyte (mg/mg)			
UCa/Cr	0.10 ± 0.01	0.13 ± 0.03	0.41
UMg/Cr	0.06 ± 0.01	0.11 ± 0.03	0.10
Urine pH	6.6 ± 0.3	5.4 ± 0.3	0.04*
UPC	0.14 ± 0.02	0.15 ± 0.07	0.17
Urine specific gravity	1.030 ± 0.002	1.035 ± 0.004	0.27

Results presented as mean ± SE. SE=Standard error, UCa/Cr=Urine calcium per urine creatinine, UMg/Cr=Urine magnesium to urine creatinine, UPC=Urine protein to creatinine ratio

### Identification of urinary proteome

A total of 74 proteins were identified in the study samples, of which 55 and 68 proteins were found in the samples from the stone-free and CaOx stone groups, respectively. In both groups, 49 proteins were found, and the top 10 most abundant proteins are shown in Table-2. Meanwhile, only six proteins were found in the stone-free group (Table-3) and 19 in the CaOx stone group (Table-4).

**Table-2:** List of top 10 most abundant proteins found in both CaOx dogs and stone-free dogs.

UniProt ID	Protein description	Freq. (n = 14)	Score SEQUEST HT	UqP	Coverage (%)
P49822	Albumin	14	10633.25	83	89.6
Q862Z3	Uromodulin	14	2703.61	31	42.4
Q8MJU5	Alpha-fetoprotein	14	53.71	6	11.3
Q767J3	Deoxyribonuclease-1	14	495.35	10	36.3
P01784	Ig heavy chain V region GOM	14	304.07	2	26.3
P19006	Haptoglobin	14	1709.42	35	72.0
P09582	Arginine esterase	14	997.11	16	56.2
P02648	Apolipoprotein A-I	14	760.79	33	76.7
Q9GMY6	Pepsin A	14	330.43	7	12.7
P01785	Ig heavy chain V region MOO	14	159.41	2	23.1

Freq.=Frequency, UqP=Unique peptides

**Table-3:** List of urinary proteins found only in stone-free dogs.

UniProt ID	Protein description	Freq. (n = 7)	Score SEQUEST HT	UqP	Coverage (%)
Q71DR4	Ciliary neurotrophic factor receptor subunit alpha	5	13.44	2	6.5
P48300	Tissue alpha-L-fucosidase	3	5.29	2	4.7
P0658 vc3	Sodium/potassium-transporting ATPase subunit beta-1	2	0	2	6.9
Q2PT31	Myocilin	1	6.42	3	5.2
Q9GLD3	Transferrin receptor protein 1	1	3.29	2	2.6
P50994	Annexin A4	1	4.61	2	9.1

Freq.=Frequency, UqP=Unique peptides

**Table-4:** List of urinary proteins found only in CaOx stone-forming dogs.

UniProt ID	Protein description	Freq. (n = 7)	Score SEQUEST HT	UqP	Coverage (%)
Q8SQ41	Pepsin B	6	14.07	2	5.1
Q28894	WAP four-disulfide core domain protein 2	4	19.44	2	38.8
Q53VB8	Ferritin light chain	4	9.44	2	17.7
O18835	Beta-glucuronidase	4	4.12	4	5.4
Q9GL24	Procathepsin L	3	11.03	3	11.4
O18733	Matrix metalloproteinase-9	2	91.35	22	35.7
E2RE76	Apolipoprotein A-IV	2	14.12	4	11.1
P68213	Fibrinogen alpha chain	1	15.7	3	67.9
P81709	Lysozyme C, spleen isozyme	1	29.91	3	27.7
P18470	DLA class II histocompatibility antigen, DR-1 beta chain	1	5.58	2	10.2
P51152	Ras-related protein Rab-12	1	3.96	2	10.1
P19540	Coagulation factor IX	1	7.07	3	6.2
Q258K2	Myosin-9	1	4.7	8	4.7
Q5XNR9	Leukemia inhibitory factor receptor	1	2.08	2	3.3
Q9TSZ6	Dystroglycan	1	3.1	2	3.2
B6V8E6	Catenin beta-1	1	3.11	2	3.1
Q9GLK0	Protein-glutamine gamma-glutamyltransferase K	1	1.9	2	2.0
Q076A7	Myosin-2	1	1.78	2	1.6
Q076A6	Myosin-1	1	0	1	0.8

Freq.=Frequency, UqP=Unique peptides

### Comparison of the urine proteomes in the CaOx and the stone-free groups

When comparing the 49 proteins found in both groups, the top 10 differences in urinary proteins identified by LC-MS/MS, including their functions are represented in Table-5. Of these, the levels of eight proteins were higher, and the levels of two proteins were lower in the CaOx stone group than in the stone-free group. Only the level of thrombomodulin was significantly higher in the CaOx stone group than in the stone-free group (p < 0.01).

**Table-5:** The difference of urinary protein levels identified in stone-free and CaOx stone-former dogs.

UniProt ID	Protein description	Ratio	Molecular function	Biological process	p-value
Q5W7P8	Thrombomodulin	1.8	endothelial cell receptor	blood coagulation	0.007
P33729	Intercellular adhesion molecule 1	0.5	integrin binding	cell adhesion	0.142
P49256	Vesicular integral-membrane protein, VIP36	1.6	protein binding	protein transport	0.157
P60524	Hemoglobin subunit beta	5.0	oxygen transport	oxygen transport	0.165
Q9TSX8	Pantetheinase	2.0	hydrolase	pantothenate metabolic process	0.167
O46634	CD166 antigen	2.1	Cell adhesion molecule	immunity, cell adhesion	0.181
Q9BEA0	Pro-epidermal growth factor	0.8	growth factor	receptor signaling pathway	0.232
P01785	Ig heavy chain V region MOO	1.4	antigen binding	immunity	0.280
O97578	Dipeptidyl peptidase 1	2.2	protease	proteolysis	0.286
Q7YQC6	Heat shock 70 kDa protein 1	1.4	Chaperone	stress response	0.291

CaOx=Calcium oxalate, VIP36=Vesicular integral membrane protein, Ig=Immunoglobulin

## Discussion

In this study, we investigated the urinary proteomic profiles between the stone-free and CaOx stone groups with the hypercalciuric condition. All dogs had serum creatinine levels within the normal reference range, which was used to assess renal function. The breed, sex, and age were matched between the groups to reduce other possible factors that could affect urinary protein excretion. The urine-specific gravity was not different between the CaOx stone group and the stone-free group. The CaOx stone group had lower urine pH than the stone-free group as previously reported [18], but not other parameters [19]. Moreover, the CaOx stone group receiving a diet formulated for urine acidity had 3 times increased risk for CaOx stone formation [20]. However, various factors, including diet, age, and other concurrent conditions might affect urine acidity.

For the urinary proteome, 74 proteins were identified in study samples using SDS-PAGE followed by LC-MS/MS, which were quite different from the study in healthy dogs [21] that could be from the difference in the population (hypercalciuric dogs vs. normal healthy) and method of sample analysis. For example, we did not separate the exosome fraction from the soluble fraction. While 49 proteins were expressed in hypercalciuric CaOx stone group and stone-free group, some of the identified proteins were comparable such as albumin, uromodulin, and immunoglobulin. Some were unique, including thrombomodulin, the 36 kDa vesicular integral membrane protein (VIP36), or pantetheinase. The difference in the levels of urinary protein may indicate the pathophysiology of CaOx stone formation. From the functional analysis, several urinary proteins play a role in blood coagulation, inflammatory process, response to oxidative stress, and immunity although specific proteins may be variable.

In our study, the levels of a protein involved in blood coagulation, such as thrombomodulin, were significantly increased in the hypercalciuric CaOx dogs. This glycoprotein is expressed primarily by endothelial cells. Thrombomodulin plays a pivotal role in the anticoagulant process by the binding of thrombin and activation of protein C, resulting in the inhibition of the coagulation cascade [22]. Increased levels of thrombomodulin were associated with an increased risk of bleeding in humans who received warfarin treatment [23, 24]. In addition, hemoglobin subunit beta levels were increased in CaOx dogs. This protein was identified in the human CaOx renal stone matrix [25]. It increased during intravascular hemolysis and coagulation [26]. A previous study showed that other proteins involved in blood coagulation, such as fibrinogen and vitronectin, in the stone matrix and urine of patients with CaOx stone formation [12]. Based on pathological conditions, stone causes renal and urinary tract injuries resulting in local bleeding and activation of the coagulation pathway, as previously suggested [12].

Besides the role of CaOx stones in the coagulation process, the overproduction of reactive oxygen species occurred. The adhesion of CaOx crystals to renal tubular epithelial cells can modulate ROS overproduction, leading to the production of molecular chaperones in response to oxidative stress, which causes renal cell injury [8]. Our data were consistent with other proteomic reports that several markers of oxidative stress were detected in the urine of patients with CaOx nephrolithiasis [12]. A previous study showed that oxidative stress may occur only in dogs with CaOx uroliths, resulting in decreased red blood cell catalase activity [27]. This study showed the proteins related to oxidative stress, such as thrombomodulin and pantetheinase. Thrombomodulin was reported to be an endothelial dysfunction marker and oxidative stress marker and is associated with chronic kidney disease in children [28], whereas pantetheinase, which is encoded by vascular noninflammatory molecule-1 or vanin-1 gene and expressed mainly in renal tubular epithelial cells, intestine, and liver, plays a role in the production of potent antioxidants [29]. Previous study has indicated the role of vanin-1 in oxidative stress. For example, vanin-1 was upregulated during oxidative stress in renal ischemia–reperfusion rats [30]. By contrast, the study in mice exposed to oxidative stress and had intestinal damage by irradiation revealed that vanin-1-knockout mice were tolerant of oxidative stress, which could result in increased stores of glutathione [31].

In addition to the role of thrombomodulin and pantetheinase on oxidative stress, they are also involved in the inflammatory process and immune response. Studies have reported the anti-inflammatory effect of thrombomodulin by inhibiting leukocyte infiltration and suppressing glomerular complement activation in diabetic mice [32, 33]. The inhibition of pro-inflammatory and profibrosis of thrombin through the protease-activated receptor pathway is considered an important protective mechanism of thrombomodulin in the kidney [34]. Pantetheinase and vanin-1 also play a role in the inflammatory process that leads to acute kidney injury. For example, urinary and serum vanin-1 were upregulated in inflammation induced by ethylene glycol-induced kidney injury [35]. Increased levels of urinary vanin-1 associated with acute kidney injury were observed in patients with upper urinary tract obstruction and decreased at 4 weeks after intervention [36].

Immunoglobulins are commonly abundant in the urine and stone matrix of patients with CaOx renal stones [12, 37]. From our results, the level of intercellular adhesion molecule-1, which is one of the immunoglobulin molecules that interact with integrin found in leukocytes, was lower in the CaOx stone group than in the stone-free group. This glycoprotein may play a role in the pathogenesis of kidney stones and several forms of nephropathy in humans [38, 39]. VIP36 has been incorporated into the kidney stone matrix of humans [40, 41]. According to reports, this glycoprotein is involved in calcium transport [42, 43], secretory pathway, glycoprotein transport, and sorting [44].

According to the location of the stones in the urinary tract, kidney stones are the most prevalent in humans, which result in the obstruction of urine flow and chronic inflammation and have been associated with reduced kidney function and chronic kidney disease [45, 46]. While kidney stones are common in dogs, lower urinary tract stones pose more of a clinical problem [47]. Dogs are more likely to develop uroliths in the lower than in the upper urinary tract and this may have less effect on kidney disease progression [48].

This study has a few limitations. A small number of dogs were enrolled in this study. The effect of sex hormones in urine protein excretion could not be ruled out because each group only had one female dog. Other factors such as dietary difference, history of cystotomy, hematuria, and timing of sample collection after cystotomy may have impacted the urine proteome changes between the CaOx stone and stone-free groups.

## Conclusion

In this study, hypercalciuric CaOx dogs showed increased level of urinary proteins, including thrombomodulin, which may be used as candidates for urinary tract injury and lead to an enhanced understanding of the pathophysiology of CaOx urolithiasis in dogs. Further investigations are needed to validate the candidate proteins as urinary injury biomarkers in patients with CaOx stones and the significance of these proteins in kidney function.

## Authors’ Contributions

CB, SC, KA, TD, NC, and TP: Contributed to conception and design of the study, performed statistical analysis, and wrote the manuscript. SC, TD, NC, TP, and NK: Contributed to data collection and laboratory analysis. SC, CB, and KA: Drafted the manuscript. SC, CB, KA, TD, and TP: Revised the manuscript. All authors have read and approved the final manuscript.
